# Therapeutic Antiaging Strategies

**DOI:** 10.3390/biomedicines10102515

**Published:** 2022-10-08

**Authors:** Shailendra Kumar Mishra, Vyshnavy Balendra, Josephine Esposto, Ahmad A. Obaid, Ricardo B. Maccioni, Niraj Kumar Jha, George Perry, Mahmoud Moustafa, Mohammed Al-Shehri, Mahendra P. Singh, Anmar Anwar Khan, Emanuel Vamanu, Sandeep Kumar Singh

**Affiliations:** 1 Indian Scientific Education and Technology Foundation, Lucknow 226002, India; 2 Saint James School of Medicine, Park Ridge, IL 60068, USA; 3 Department of Environmental and Life Sciences, Trent University, Peterborough, ON K9L 0G2, Canada; 4 Laboratory Medicine Department, Faculty of Applied Medical Sciences, Umm Al-Qura University, Makkah 21955, Saudi Arabia; 5 Laboratory of Neurosciences and Functional Medicine, International Center for Biomedicine, University of Chile, Santiago 7750000, Chile; 6 Department of Biotechnology, School of Engineering & Technology (SET), Sharda University, Greater Noida 201310, India; 7 Department of Biotechnology, School of Applied & Life Sciences, Uttranchal University, Dehradun 248007, India; 8 Department of Biotechnology, Engineering and Food Technology, Chandigarh University, Mohali 140413, India; 9 Department of Biology, The University of Texas at San Antonio (UTSA), San Antonio, TX 78249, USA; 10 Department of Biology, College of Science, King Khalid University, Abha 9004, Saudi Arabia; 11 Department of Botany and Microbiology, Faculty of Science, South Valley University, Qena 83523, Egypt; 12 School of Bioengineering and Biosciences, Lovely Professional University, Phagwara 144411, India; 13 Laboratory Medicine Department, College of Applied Medical Sciences, Umm Al-Qura University, Makkah 78249, Saudi Arabia; 14 Faculty of Biotechnology, University of Agricultural Sciences and Veterinary Medicine, 011464 Bucharest, Romania

**Keywords:** aging, hallmarks, risk factors, therapeutic agent, antiaging strategies

## Abstract

Aging constitutes progressive physiological changes in an organism. These changes alter the normal biological functions, such as the ability to manage metabolic stress, and eventually lead to cellular senescence. The process itself is characterized by nine hallmarks: genomic instability, telomere attrition, epigenetic alterations, loss of proteostasis, deregulated nutrient sensing, mitochondrial dysfunction, cellular senescence, stem cell exhaustion, and altered intercellular communication. These hallmarks are risk factors for pathologies, such as cardiovascular diseases, neurodegenerative diseases, and cancer. Emerging evidence has been focused on examining the genetic pathways and biological processes in organisms surrounding these nine hallmarks. From here, the therapeutic approaches can be addressed in hopes of slowing the progression of aging. In this review, data have been collected on the hallmarks and their relative contributions to aging and supplemented with in vitro and in vivo antiaging research experiments. It is the intention of this article to highlight the most important antiaging strategies that researchers have proposed, including preventive measures, systemic therapeutic agents, and invasive procedures, that will promote healthy aging and increase human life expectancy with decreased side effects.

## 1. Introduction

**Figure 1:**
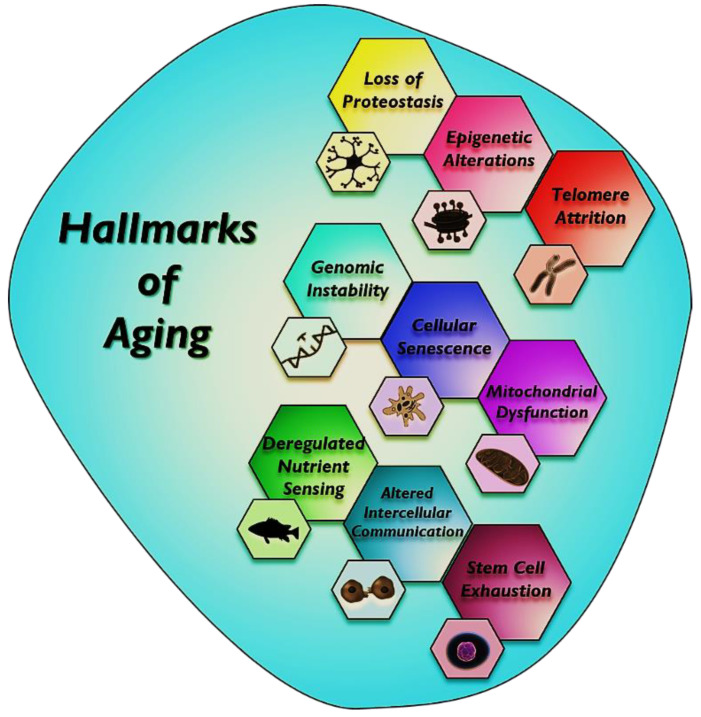
Hallmarks of aging. This scheme identifies the nine hallmarks briefly described in this review: loss of proteostasis, epigenetic alterations, telomere attrition, genomic instability, cellular senescence, mitochondrial dysfunction, deregulated nutrient sensing, altered intercellular communication, and stem cell exhaustion.

Aging is depicted by a gradual functional decline causing the progressive deterioration that occurs heterogeneously across multiple organs, leading to organ dysfunction in mammals. It is a major risk factor for common chronic diseases, such as heart disease, cancer, diabetes, and Alzheimer’s diseases [1]. In recent years, aging research has been geared toward advancing upon knowledge established on the nine hallmarks of aging at the cellular and molecular levels [2]. Several hallmarks of aging are as follows: (i) genomic instability. high frequency of mutations within the genome of the cell; (ii) telomere attrition, i.e., protective caps going to end with each subsequent cell division; (iii) epigenetic alterations, i.e., DNA methylation, histone modification, and chromatin remodeling that change in gene expression; (iv) loss of proteostasis, i.e., folding, chaperoning, and maintenance of protein function collapses; (v) deregulation of nutrient sensing, i.e., reduction in the function of IGF-1, mTOR, sitrulins, and AMPK that regulate the metabolism; (vi) mitochondrial dysfunction, i.e., reduced efficiency of oxidative phosphorylation and reduction in the production of ATP; (vii) cellular senescence, i.e., ended the activation of p53 and the cyclin-dependent kinase (CDK) inhibitor p16 due to a variety of mechanisms, such as telomere shortening, forms of genotoxic stress, mitogens, and inflammatory cytokines; (viii) stem cell exhaustion, i.e., reduction in stem cell activity and altered intercellular communication, and (ix) the burden of senescent cells (Figure 1).

Recently, scientific achievements have been focused on producing effective antiaging therapeutics that have dramatically improved human life expectancy. Many studies on animal models looking at genetics and dietary and pharmacological interventions have shown an enhanced lifespan [3,4,5]. Other studies have examined antiaging strategies, such as enhancement of autophagy, elimination of senescent cells, transfusion of young blood, intermittent fasting, stem cell therapy, physical exercise, adult neurogenesis boost, and antioxidant and herbal intakes, which have emerged in recent years [6]. In humans, evidence has been accumulated on the styles of life in increasing the lifespan [7].

In this review, we have attempted to collect various in vitro and in vivo research experiments for antiaging approaches based on the major hallmarks of aging. Here, we describe antiaging strategies such as telomere reactivation, epigenetic drugs, activation of chaperons and proteolytic systems, and dietary restriction. Mitophagy and the clearance of senescent cells, etc. will be beneficial in understanding the unique approaches for successful aging and to extend healthy lifespans.

## 2. Anti-Inflammatory Drugs Used as an Antiaging Approach

Chronic inflammation is one of the major contributors to age-associated diseases and aging and disrupts the normal functioning of tissues [1,8,9]. Increased activity of proinflammatory pathways accompanies inflammation with age [10,11]. Serum concentrations of proinflammatory cytokines (IL-1, IL-2, IL-6, IL-8, IL-12, IL-15, IL-17, IL-18, IL-22, IL-23, tumor necrosis factor-α, and interferon-γ) are significantly increased in normal process aging compared with younger individuals in a normal stage [12,13,14]. A chronic proinflammatory status is a pervasive feature of aging, and increased systemic inflammation is closely associated with aging and age-related diseases [15,16]. The term “inflammaging” is used to describe aging induced by chronic and persistent inflammation. Anti-inflammatory agents block certain substances in the body that cause inflammation, and various studies have shown that anti-inflammatory agents are linked to antiaging [17]. The most important drivers of age-dependent inflammation are derived at the cellular and molecular levels. In a cell, the proinflammatory senescence-associated secretory phenotype (SASP) is associated with cellular senescence that is triggered by agents such as radiation and viruses and by continuous exposure to cellular debris [18] and cellular senescence [19]. In a molecule, ROS (reactive oxygen species) and other agents can trigger inflammatory DNA damage responses that affect DNA and telomeres [20] and activate the inflammasomes and NF-kB pathway [21]. Inhibition of the inflammatory processes by genetic and pharmacological intervention is considered an effective and verified antiaging strategy [2]. Nonsteroidal anti-inflammatory drugs (NSAIDs) not only prevent certain age-associated features but also increase the lifespan in various model organisms, such as yeasts [22], nematodes [23], mice [24,25], and flies [22]; however, their effectiveness for neurodegenerative disorders (Alzheimer’s disease and Huntington’s disease [26]) is not clear, and there is a search for anti-inflammatory bioactive compounds [27,28,29,30,31]. Anti-inflammatory drugs might be considered to have great potential for extending the lifespan. In some studies, spermidine (polyamines) and their action on the expression of pro- and anti-inflammatory cytokines can directly reduce inflammation and indirectly alter inflammation and cell growth by the action of autophagy [32]. Spermidine has been reported to slow down aging due to its antiaging effects [33]. Aspirin, a potent anti-inflammatory and antioxidant compound, may affect oxidant production and cytokine responses and block glycoxidation reactions that protect against oxidative stress, as well as extend the lifespan of *Caenorhabditis elegans* and mice [34,35,36]. Ibuprofen (NSAID) has been shown to reduce the risk of age-related pathologies and increase the lifespan of *Saccharomyces cerevisiae*, *Caenorhabditis elegans,* and *Drosophila melanogaster* [37,38]. A novel NSAID, M2000, could modify oxidative stress pathways by lowering the expression levels of the SOD_2,_ GST, iNOS, and MPO genes and reduce the risk of inflammatory diseases through its immunosuppressive effects, with no adverse side effects on the enzymatic and nonenzymatic determinants [39]. This can be recommended as an antiaging drug. MAAs (mycosporine-like amino acids), such as M2G (mycosporine-2-glycine), exhibit antioxidant, anti-inflammatory, anti-protein-glycation, and collagenase inhibition activities and show the ability to protect DNA against UV damage [40,41]. Many nutraceuticals (apigenin, quercetin, kaempferol, naringenin, catechins, epigallocatechin, genistein, cyanidin, resveratrol, etc.) and functional foods possess antioxidant activity that might play an important role in delaying aging and be effective in various human neurodegenerative diseases [30,42,43,44,45].

## 3. Antioxidant Activity

Phytochemicals such as phenolic acids and flavonoids have antioxidant activity, which acts by scavenging free radicals and increasing the levels of antioxidant enzymes in plasma [46]. The function of a primary antioxidant enzyme is to protect organisms from the damaging effects of superoxide radicals, which are quickened by their dismutation into hydrogen peroxide and oxygen [47]. Several studies have confirmed that quercetin is a strong antioxidant that accumulates in nematodes and displays reactive oxygen species (ROS) scavenging activity and has been demonstrated to have a positive effect on longevity and stress resistance in various animal models [48,49,50,51]. Many studies have demonstrated that NSAIDs have antioxidant activity that is mediated by free radical scavenging and antioxidant enzyme activation [52]. The antioxidant activity of NSAIDs has been witnessed in membranes, cells, and at the organismal level [52,53,54].

## 4. Telomere Reactivation

Telomeres are conserved microsatellite repeats TTAGGG that protect the ends of chromosomes from DNA breakage and prevent DNA end-joining, recombination, and DNA repair [55]. DNA polymerases are incapable of fully replicating the linear chromosomes owing to end replication in somatic cells, and telomeres become gradually shortened after each cell division [56]. This shortening of telomeres is usually fulfilled by the telomerase enzyme, but most somatic cells and adult stem cells do not express enough telomerase to compensate for the telomere length that leads to entering ‘replicative senescence’, which might be followed by cell death [57,58,59]. Telomere shortening occurs during normal aging and is an important biomarker of aging and longevity that is influenced by several factors, such as genetics, epigenetics, and environments [60,61,62,63]. It is also associated with many age-related diseases, such as osteoarthritis, atherosclerosis, coronary heart disease, and atrial fibrillation [64,65,66]. Several studies have reported that aging can be inhibited by the overexpression of telomerase; however, it can enhance tumorigenesis [67,68,69]. A telomerase activator, telomerase expression activator, and telomerase gene therapy have been developed as telomerase-based antiaging strategies in recent years. TA-65 is an extract of a Chinese plant (*Astragalus membranaceus),* a telomerase activator that can restore telomere length without cancer occurrence and improve age-related indicators, including glucose tolerance, bone health, and skin quality [70]. Additionally, some studies found that TERT transcription activator and sex hormones are directly involved in activating telomerase, which rescued telomere shortening and enhanced the lifespan [71,72]. Evidence has suggested that the reactivation of telomerase expression by using a gene therapy approach is the best example of the lifespan extension of mice and delay aging without cancer occurrence [73]. A recent study has found that Metadichol, a telomerase activator, is used to overcome organ failure by enriching cells with telomerase and is a safer alternative [74]. Another study explained that natural compounds such as 08AGTLF *(Centella asiatica),* Nutrient 4 (*Astragalus*), TA-65 (*Astragalus membranaceus*), OA (oleanolic acid), and MA (maslinic acid); and Nutrients 1, 2, and 3 have telomerase activation, and among all, 08AGTLF has the greatest potential to activate telomerase [75]. The impact of telomeric length on humans has been evidenced by the fact that the expression of telomerase in normal cells may extend a healthy lifespan; however, inhibition of telomerase in cancer cells may be a viable target for anticancer therapeutics [76].

## 5. Antiaging Approaches Using Epigenetic Drugs

**Figure 2:**
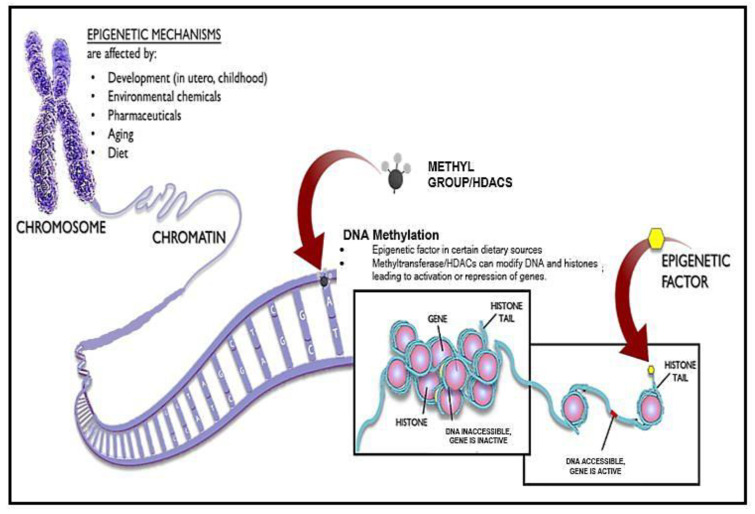
Epigenetic Mechanisms via DNA Methylation and histone modification. Epigenetics can be altered via developmental mechanisms, environmental chemicals, pharmaceuticals, aging, and diet. Some dietary sources can lead to a direct production of DNA methylation, allowing for the overexpression or repression of genes, thereby increasing the aging process.

The effects of chromatin on aging are probably complex and bidirectional. Chromatin remodeling appears to counter aging and age-associated diseases and extend organismal lifespan [77]. Chromatin is intensely altered during aging owing to the decreased level of histone proteins and adequate changes in histone modification that were found in recent studies in budding yeast and human fibroblast cells [78]. Elevating histone expression, reducing H4 K16 acetylation, reducing H3 N-terminal acetylation, inactivating the HDAC Rpd3, and inactivating the H3K4 methylase might be capable of extending lifespan or reverting the aged phenotype to a more youthful state of chromatin [79]. These epigenetic factors are influenced by diet, lifestyle and exogenous stress, which raises the possibility of enhancing age-related cellular dysfunction [80,81]. Several studies revealed age-associated changes in DNA methylation patterns leading to a global reduction in DNA methylation [82] (Figure 2); site-specific hypermethylation, specifically at CpG islands and polycomb target sites [83]; site-specific hypomethylation specifically at gene-poor regions, tissue-specific promoters, and polycomb protein regions; and hypermethylation in different tissues but hypomethylation, to be more tissue specific [84,85]. These changes accumulate gradually; such changes are indicative of the aging process and strongly associated with epigenetic changes such as replicative senescence [86]. Diet, lifestyle, environmental interventions, and inhibitors of epigenetic enzymes have proven to be effective in promoting longevity, as seen in various experiments [87]. Natural substances such as spermidine and resveratrol have been found to lead to deacetylation of chromatin, indicating that it has the potential to extend the lifespan in humans [88]. Many studies report that HDAC (histone deacetylase) inhibitors show evidence as an antiaging strategy [89]. HDAC inhibitors have the potential to reverse the aging process that allows healthy aging [90].

## 6. Activation of Chaperons and the Proteolytic System against Aging

Loss of proteostasis is a common feature of aging that leads to protein aggregation, unfolding, oxidative damage, posttranslational modification, and an altered rate of protein turnover and, ultimately, to cellular dysfunction [91,92,93,94,95]. Two proteolytic systems—the ubiquitin–proteasome system (UPS) and autophagy–lysosome system (ALS)—and chaperones play a major role in maintaining proteostasis [96]. The alteration or deterioration of these pathways impairs normal cell functioning and cell physiology, causing aging [2]. Many studies have found proteostasis changes with age owing to reduced activity of heat-shocked protein chaperones [97,98]. To compensate for this decline, increasing the chaperone protein level has been shown to beneficially impact longevity in worms and flies [99,100,101,102]. A study reported that the aggregation of Hsp104, a chaperone, has been associated with aging and has increased the lifespan in *Saccharomyces cerevisiae* [103,104,105]. The UPS and ALS systems are the main proteolytic systems that influence the cellular fate and aging process [106,107]. The availability of the chaperone is extremely compromised in aged cells in which the proteostasis collapses by decreased G1-cyclin function that causes an irreversible arrest in G1, configuring a molecular pathway claiming proteostasis deterioration leads to cell senescence [108]. Promoting proteasomal activity via overexpression of the proteasomal β5 subunit either in *Caenorhabditis elegans* [109] or in human fibroblast [110] and the overexpression of Rpn11 in *Drosophila melanogaster* [111] increases the lifespan and stress resistance. The compound 18α-glycyrrhetinic acid and loss of IGF (insulin-like growth factor) signaling due to mutated daf-2 induce proteasomal activation and extend the lifespan of *Caenorhabditis elegans* [112]. Spermidine, metformin, rapamycin, and resveratrol are pharmaceutical approaches well-known to activate the autophagy system [113]. In a recent study, it was found that minocycline, JZL184, monorden, and paxilline directly targeted the 18S rRNA/ribosome, FAAH-4, Hsp90, and the SLO-1 BK channel, significantly increasing the lifespan of *Caenorhabditis elegans* [114]. The proteostatic system governs the synthesis and conformation of target proteins, and the ubiquitin—proteasome system and autophagy act as the main scavengers of misfolded or excessive proteins. The main cause of Alzheimer’s disease is the accumulation of misfolded proteins as Aβ plaques and tau aggregates owing to dysregulation of proteostasis, which contributes to the accumulation of proteotoxins in Alzheimer’s disease [115]. It has been reported that a decrease in the efficiency of the autophagy and ubiquitin—proteasome systems might lead to aging and neurodegenerative diseases such as AD, PD, and ALS [116]. The prion diseases in mammals are related to altered versions of PrP^c^ (cellular), which is a key component of the infectious agent responsible for transmission, and the disease-associated version of PrP^c^ can be partially resistant to the protease–digestion system, designated PrP^sc^ (scrapie) [117]. Various cellular components, predominantly chaperones such as Hsp104, Hsp40s, HSP42, and HSP70s, can lead to the curing of yeast prions by their deficiency or overproduction. These studies have revealed the requirements for prion propagation and conditions that affect prion stability, which have led to the discovery of anti-prion systems. Btn2p, a component of the yeast anti-prion system, has the aggregate-sequestering abilities; it can cure an artificial and natural yeast prion, and works on a variety of non-prion aggregates as well [118]. This system might be advantageous for neurodegenerative diseases that result from aggregates of proteo-toxins.

## 7. Mitophagy Activators as an Antiaging Approach

**Figure 3:**
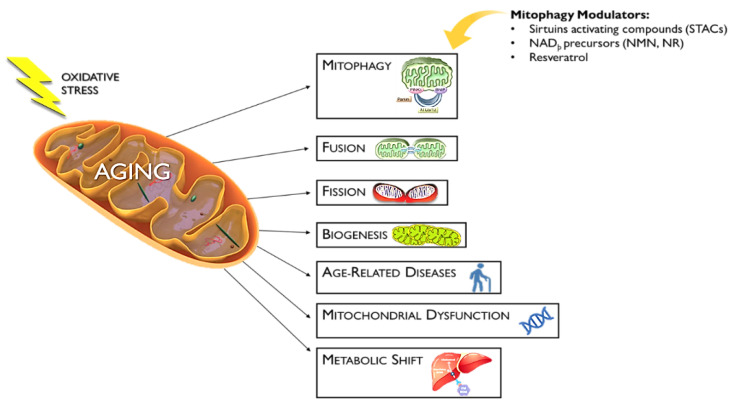
Mitophagy Modulators Against Mitochondrial Dysfunction. Several factors contribute to the aging process via the mitochondria, including mitophagy, mitochondrial fusion and fission, biogenesis, age-related diseases (i.e., AD and PD), mitochondrial dysfunction in general, and metabolic shifts. STACs, NMN, NR, and resveratrol are compounds that act against the aging process, directly targeting mitophagy and preventing the selective degradation of healthy mitochondria.

Mitochondrial dysfunction is one of the hallmarks of aging [2] that is associated with aging and is also involved in the development of many neurodegenerative diseases [119]. Mitochondrial dysfunction is caused by a defect in mitophagy that has been associated with aging and other diseases such as metabolic disorders, cancer, senescence, inflammation, and genomic instability [120]. Mitochondrial health plays an important role in the aging process that can assist in the therapeutic approach toward longevity [121]. Mitophagy could turn to a pro-death pathway due to excessive superoxide stress that leads to accelerated aging [122]. Parkin is a key protein in mitophagy [123] and is overexpressed, leading to a decreased lifespan in Drosophila [124,125]. Mitochondrial health depends on inflammation, because many inflammatory pathologies have been associated with mitochondrial defects [126]. Studies report that many natural products have the potential to act as antiaging strategies [127]. Many inducers, such as sirtuin-activating compounds (STACs), NAD_þ_ precursors (NMN and NR), and resveratrol, have been shown to modulate mitophagy and repair mitochondrial functions [128,129,130] (Figure 3).

Resveratrol (3,5,4-trihydroxystilbene) is a natural polyphenol that can induce autophagy and mitophagy by different mechanisms, such as activation of AMPK and SIRT1; induction of P38 MAPK; suppression of mTOR and p70S6K; and upregulation of *eNOS*, *GABARAP*, *LC3B,* and *ATG3* genes, which is a beneficial compound for extending the lifespan [131,132,133,134,135,136,137,138].

Some natural compounds, such as spermidine and urolithin, preserve mitochondrial function and extend longevity through mitophagy induction, which has been demonstrated in several model organisms, such as yeast, nematodes, flies, and mice [113,139,140,141]. Moreover, tomatidine is a natural compound that stimulates the elimination of defective mitochondria, leading to increased longevity and enhanced muscular function in nematodes and mice [142]. Some studies reveal that antibiotics severely affect mitochondrial homeostasis [143]. Actinomycin and doxycycline interrupt energy metabolism and facilitate mitophagy in mammalian cells [144,145]. The mitochondria generates most cellular ROS, but mtDNA is comparatively unprotected from ROS damage due to a lack of histone proteins and a lack of robust mtDNA repair mechanisms. A decline in mitochondrial energy metabolism and accumulation of mtDNA mutations in tissue cells are important contributors to human aging, and some type of mtDNA mutations are leading to the development of cancer [146]. A study showed that deficiency and/or dysfunction in many of the nuclear-encoded mtDNA replicative proteins, such as Pol γ, PolG2, Twinkle, TFAM, MGME1, and RNase H1, are responsible for the development of age-related diseases and/or phenotypes both in vitro and in vivo [147].

## 8. Inhibition of mTOR and Insulin/IGF-1 Signaling (IIS) as an Antiaging Approach

mTOR promotes cell growth by either promoting protein synthesis or inhibiting autophagy activity. It is involved in senescence-associated phenotypes. The mTOR pathway acts as a nutrient sensor through regulation of mitochondrial biogenesis [148], regulation of mitophagy [149], promotion of the secretory phenotype of senescent cells [150], and inhibition of stem cell senescence [151,152]. Many studies have demonstrated that a reduction of mTOR signaling is associated with extension of the lifespan in *Caenorhabditis elegans* [153], *Drosophila melanogaster* [154,155], *Saccharomyces cerevisiae* [156,157], and *Mus musculus* [158]. Thus, it is the most observed target for the study of pharmacological treatments. Pharmacological inhibition of mTOR by rapamycin confirms that it can be observed to slow down aging in yeast (*S. cerevisiae*) [159], *Drosophila melanogaster* [154,160,161,162,163,164], worm (*C. elegans*) [165], and mice (*M. musculus*) [166,167,168,169,170,171,172,173,174,175,176,177,178,179,180,181,182,183,184,185,186,187]. It has also been seen to delay age-related diseases in different species including humans [188,189,190,191,192,193,194,195,196,197,198,199,200]. mTORC1 has two substrates, S6 kinase and 4E-BP1, and both are linked to longevity through the reduction of S6 kinase, which promotes longevity [201]. Another nutrient-sensing pathway is the insulin/insulin-like growth factor 1 (IGF-1) signaling (IIS) network, which is a major determinant of longevity important to regulate the lifespan in various species, such as *C. elegans* [202], *D. melanogaster* [203,204], and *M. musculus* [205,206]. It has been shown that suppression of the IIS/mTOR pathways is involved in prolonging the lifespan but also delays the onset of age-related pathologies in numerous organisms, such as yeast, fruit flies, nematodes, mice, rats, and primates [207,208,209]. In recent years, studies have reported that celecoxib (a nonsteroidal anti-inflammatory drug), recombinant BTI (rBTI-mimicking restriction), and the ethyl acetate fraction of *Ribes fasciculatum* (ERF) downregulate the insulin/IGF-1 signaling cascade and extend the lifespan in *Caenorhabditis elegans* [23,210]. Recently, a study demonstrated that hypo-taurine, an antioxidant, induces the lifespan in *Caenorhabditis elegans* by regulating the insulin/IGF-1 signaling (IIS) pathway [211,212].

## 9. Activation of AMPK and Sirtuin Signaling as an Antiaging Approach

5′adenosine monophosphate-activated protein kinase (AMPK) is a master regulator of energy metabolism in the cell that plays a critical role in regulating health and longevity [213]. AMPK is a serine/threonine protein kinase that is a highly conserved sensor that increases the levels of AMP and ADP [214,215]. Sirtuins (SIRT1 to SIRT7), also known as histone deacetylases (HDACs), delay cellular senescence and promote longevity in various species through increased expression [216,217,218,219,220,221]. Many FDA-approved drugs, such as biguanides, thiazolidinediones, glucagon-like peptide-1 receptor agonists, salicylates and resveratrol, and 5-aminoimidazole-4-carboxamide riboside (AICAR), have AMPK-activating properties [222,223]. Resveratrol (3,5,40-trihydroxystilbene) is a natural activator that increases the production of synthetic SIRT1 activators, which are more potent, soluble and bioavailable [136,224]. Some synthetic activators, such as SRT1720 (imidazothiazoles), thiazolopyridine, benzimidazole, bridged ureas, cilostazol, paeonol, statins, hydrogen sulfide, persimmon, and SRT2104 also extend the lifespan in mice and protect cells against age-related changes [225,226,227,228,229,230,231,232]. Moreover, natural compounds such as quercetin, proanthocyanidins, fisetin, catechins, kaempferol, and butein have been reported to have antiaging properties [233]. Metformin activates AAK-2/AMPK and induces autophagy through AMPK, which is pro-longevity in nematodes, Drosophila, rats, and mice [234,235,236,237,238,239,240,241]. Oligonol is an antioxidant polyphenolic compound with an anti-inflammatory property that activates the autophagy pathway and phosphorylation of AMPK in *C. elegans* [242].

## 10. Clearance of Senescent Cells

Cellular senescence is a central component of aging that leads to cell cycle arrest in a damaged cell by preventing the cell from promulgating further damaged tissues [243,244,245]. Senescent cells accumulate with age and promote aging and age-associated pathologies [246,247,248,249]. To extend the lifespan, senescent cells need to be decreased or suppressed through the senolytics approach. There are many senolytics that have been reported to induce apoptosis of senescent cells and reduce the expression of senescence markers, including a combination of dasatinib and quercetin, BCL2 family inhibitors, SCAPs, FOXO4, and piperlongumine [250,251,252,253,254,255,256,257]. Moreover, HSP90 has been identified as a novel senolytics that can induce apoptosis of senescent cells to improve the health span [258]. There are several senomorphic drugs that suppress markers of senescence or their secretory phenotype, including inhibitors of IkB kinase (IKK) and nuclear factor (NF), inhibitors of free radical scavengers and the Janus kinase (JAK) pathway. Rapamycin acts as a senomorphic agent to reduce the SASP, and ABT263 inhibits the antiapoptotic proteins BCL-2 and BCL-XL [259,260,261]. In recent years, two well-known antibiotics, azithromycin and roxithromycin, had senolytic activity to target senescent cells [262]. Curcumin is an ayurvedic formulation that has antisenescence properties and can modulate cellular senescence by activating the sirtuins and AMPK pathways [263].

## 11. Stem Cell-Based Therapies

Stem cell exhaustion (decline in stem cell number and function) is one of the causes of aging that is observed in all tissues and organs and is maintained by adult stem cells through repair and regeneration during life [264,265]. There is a loss of adult stem cells such as hematopoietic stem cells (HPSCs), which respond to stress and differentiation, and HPSCs are decreased by the hyperactivation of the mechanistic target of rapamycin complex 1 and the disruption of 5′ adenosine monophosphate-activated protein kinase (AMPK) [266,267]. It has been reported that the accumulation of DNA damage and increased levels of p16^INK4a^ have been closely linked to a decline in stem cell populations with aging [268]. Age-associated phenotypes could be restored by the induction of stem cell rejuvenation [269,270,271]. Reprogramming aged hematopoietic stem cells (HPSCs) to a pluripotent state and inducing pluripotent stem cells (iPSCs) from aged human fibroblasts into NSCs are alternate options [272,273]. Metformin is an FDA-approved drug that can induce stem cell rejuvenation in adult stem cell therapy [274]. Studies have proposed that using AMPK activators such as 5-aminoimidazole-4-carboxamide ribonucleotide, A769662, metformin, and oxidized nicotinamide adenine dinucleotide (NAD^+^) can be used in stem cell-based transplantation therapies with better results [275]. A study revealed that resveratrol-induced SIRT1 activation promotes the self-renewal of human embryonic stem cells (hESCs) [276]. A recent study reported that the administration of caloric restriction (CR) could enhance stem cell proliferation by regulating niche cells and reducing the major energy metabolic pathways that prevent stem cell aging [277].

## 12. Role of the Microbiome in Aging and Potential Antiaging Therapeutics

The evidence highlighted that microbiome composition may affect the rate of aging [278,279], although no evidence has been found that microbiota composition harshly changes the chronological threshold or age; rather, these changes proceed gradually with time [280]. Between the host and intestinal microbiota, the rate of age-related deterioration is strongly influenced by particular factors such as age-associated alterations in lifestyle, nutrition, frailty, and inflammation [281]. A study reported that the microbial composition is highly similar in young adults and seventy-year-old people but significantly differs in centenarians [280]. This difference was associated with the enrichment in opportunistic proinflammatory bacteria known as pathobionts, which are generally present in adult gut ecosystems in low numbers. The microbiota may favorably affect the host health and aging processes using prebiotics and probiotics that have been shown to be efficient in preventing particular pathological conditions, such as the suppression of inappropriate chronic inflammation in elderly populations. It includes a decrease in the synthesis of proinflammatory cytokines such as interleukins (IL-6, IL-8, IL-10, and TNF) and, thus, an increase in the levels of activated lymphocytes and natural killer cells and phagocytic activity [282]. Some other effects of microbiota, including the regulation of host fat deposition and metabolism [283], prevention of insulin resistance [284], degradation of nondigestible carbohydrates, enhancement of antioxidant activity, production of vitamin B and conjugated linoleic acids [285], and improved maintenance of mucosal barrier integrity and immune homeostasis [286]. Several research findings suggest that direct modulation of the gut microbiome might be beneficial to be applied in treating age-related disorders [287] and considered to be a novel potential therapeutic for healthy aging [288]. Some studies suggest that the intake of functional foods such as prebiotics, probiotics, or synbiotics might be an effective strategy to counter natural aging through modifying the gut microbiota of the elderly population [289].

## 13. Role of Noncoding RNAs in Aging and Their Potential Therapeutics

Long noncoding RNAs (lncRNAs) can affect key cellular processes, such as proliferation, differentiation, quiescence, senescence, the cellular response to stress and immune agents, and other cellular functions related to the biology of aging. LncRNAs have the ability to modulate gene expression patterns at the transcriptional, posttranscriptional, and posttranslational levels [290,291,292]. The changes in the subsets of expressed proteins are responsible for the aging traits. LncRNAs can modulate protein expression patterns by controlling gene transcription, mRNA stability, and protein abundance; thus, they can modulate key molecular events underlying the aging process, such as the control of telomere length, epigenetic gene expression, proteostasis, stem cell function, intercellular communication, cell proliferation, and cellular senescence [293].

## 14. Conclusions

**Figure 4:**
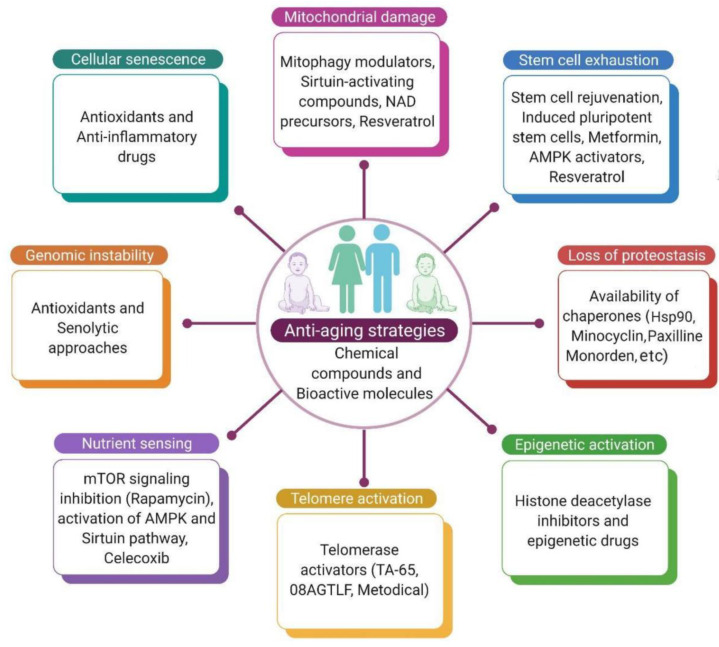
Antiaging strategies to counter aging that might extend the human lifespan based on the use of chemical compounds and bioactive molecules and their use in various approaches mentioned in the figure.

This review has attempted to explain the various antiaging strategies used based on the major hallmarks of aging. Data have been collected from scientific evidence, such as studies conducted on organisms and species targeting aging. These approaches would not only postpone chronic diseases but also prevent many age-related disorders and extend healthy lifespans. Aging and age-related diseases are due to abnormalities in normal biological pathways, such as metabolism, inflammation, growth, and protein synthesis, which also alter the rate of aging. Interestingly, modifications in the normal epigenetic pathway appear to be a major cause of chronic disorders. This review presents various protective interventions as antiaging strategies that are valuable to extend the human healthy lifespan. These include telomere reactivation, epigenetic drugs, activation of chaperons and the proteolytic system, caloric restriction, mitophagy activation, clearance of senescent cells, antioxidant and anti-inflammatory drugs, inhibition of mTOR and insulin/IGF-1 signaling (IIS), and stem cell-based therapy. Targeting senescent cells and inflammation plays a prominent role in aging, such as employing anti-inflammatory bioactive molecules in recent studies or, in some cases, nonsteroidal anti-inflammatory drugs (NSAIDs), senolytics, and SASP suppressors. To achieve adequate telomere length through telomerase activators, HDAC (histone deacetylase) inhibitors may be considered as antiaging agents. To rescue mitochondrial function and promote proteasomal activity, SIRT1 and AMPK activators and mTOR suppressors may be an effective way to combat aging. Reprogramming technology such as iPSCs and stem cell therapy, are emerging pieces of evidence for supplementing stem cells to rejuvenate tissues and organ functions. Thus, exploring the aging process at the molecular level is still a challenge, but the possibility of developing novel therapeutics by using nutraceuticals, molecular medicine, and pharmacogenomics approaches may be options for successful aging (Figure 4).

### Expert Opinion

Various scientific achievements have been focused on producing effective antiaging therapeutics that have dramatically improved human life expectancy. Many studies on animal models looking at genetics and dietary and pharmacological intervention have shown an enhanced lifespan. Thus, in this article, we have highlighted various antiaging strategies that have targeted approach-based hallmarks of aging. Researchers have proposed various strategies, such as preventive measurements, systemic therapeutic agents, and invasive procedures, that will promote healthy aging and increase human life expectancy with decreased side effects.

## Data Availability

Not applicable.
